# The impact of periodic leg movements during sleep on neurological recovery in patients with acute mild cerebral infarction

**DOI:** 10.3389/fneur.2025.1610871

**Published:** 2025-08-01

**Authors:** Shutong Sun, Yixi Zheng, Liwen Xu, Wenyi Yu, Tianyu Jing, Gang Xu, Tieyu Tang, Cheng Chu

**Affiliations:** ^1^Department of Neurology, The Affiliated Hospital of Yangzhou University, Yangzhou University, Yangzhou, China; ^2^Department of Intensive Care Unit, The Jiangyin No.3 People’s Hospital, Wuxi, China

**Keywords:** sleep periodic limb movement, acute mild cerebral infarction, neurological function, prognosis, sleep disorders

## Abstract

**Objective:**

Acute minor cerebral infarction is a common cerebrovascular disease, and its prognosis is influenced by various factors. This study aims to explore the relationship between nocturnal periodic leg movements and neurological function 3 months after acute mild cerebral infarction.

**Methods:**

A retrospective study was conducted based on hospital records, involving patients diagnosed with acute minor cerebral infarction who underwent polysomnography at Yangzhou University Affiliated Hospital from September 2023 to October 2024. Patients were followed up by phone 3 months later, and the modified Rankin Scale (mRS) was completed. Based on the scores, patients were divided into a good prognosis group (0–2 points) and a poor prognosis group (3–6 points). The correlation between sleep-related scales, polysomnography, and prognosis was analyzed using Spearman correlation analysis. Further, indicators related to prognosis (data with *p* < 0.05 in correlation analysis) along with age, hypertension, cholesterol, and radiate crown area cerebral infarction were included in a binary logistic regression analysis to assess factors affecting neurological function in patients with acute minor cerebral infarction after 3 months.

**Results:**

A total of 766 patients were included, with 203 patients in the poor prognosis group. The results indicated that patients with a history of hypertension (OR = 0.589, 95% CI = 0.401 to 0.863), higher HCY levels (OR = 1.037, 95% CI = 1.005 to 1.070), radiate crown area cerebral infarction (OR = 1.655, 95% CI = 1.150 to 2.382), longer N1% (OR = 1.032, 95% CI = 1.013 to 1.052), and higher PLMI (OR = 1.006, 95% CI = 1.001 to 1.010) are risk factors for the prognosis of Acute minor cerebral infarction patients at 3 months (*p* < 0.05).

**Conclusion:**

PLMS is an independent risk factor for impaired neurological recovery in patients with acute minor cerebral infarction. This finding suggests that systematic sleep monitoring should be conducted in clinical practice for acute minor cerebral infarction, and early identification and intervention targeting PLMS may become a new focus for improving long-term prognosis in patients.

## 1 Background

Acute minor cerebral infarction (National Institutes of Health Stroke Scale score, NIHSS ≤ 4) (1). As a common subtype of ischemic stroke, it accounts for 0061pproximately 30–40% of all stroke cases. Despite its mild early symptoms (2), studies have shown that patients may experience neurological deterioration, relapse, or residual cognitive deficits, depression, and other sequelae within 3 months of the onset of the disease (3–5), which significantly reduces the quality of life and increases the healthcare burden on society. In recent years, sleep disorders, as a common co-morbidity after stroke, have gradually been recognized as a potentially modifiable factor affecting neural repair, but their specific mechanisms of action and the value of clinical interventions need to be explored in depth. Periodic limb movements of sleep (PLMS) is a common sleep-related movement disorder that refers to rhythmic dorsiflexion of the lesser toes and ankle joints during sleep, usually accompanied by knee flexion, sometimes involving the hip and upper limbs (6). It has been shown that healthy adults are more likely to develop PLMS the older they get, and PLMS is very rare before the age of 40, after which its incidence increases dramatically (7).

The prevalence of PLMS is 7.6% in the normal population and up to 47.5% in patients with cerebrovascular disease (8, 9). As one of the common stroke subtypes, PLMS may predict disease progression and poor outcome. PLMS is involved in cardiovascular and cerebrovascular disease through several mechanisms, firstly, increased inflammation and oxidative stress, secondly, PLMS contributes to the activation of the autonomic nervous system, and lastly, metabolic derangement may also be one of the mechanisms (10). Currently, the prognostic assessment of patients with mild stroke focuses on infarct volume, vascular risk factors, and early rehabilitation interventions, while insufficient attention has been paid to sleep quality, especially PLMS (11, 12). The role played by PLMS in patients with acute minor stroke is still inconclusive, and this paper aims to investigate the effect of PLMS on neurological function in patients with acute minor stroke at 3 months.

## 2 Methods

### 2.1 Research subjects and inclusion/exclusion criteria

This study is a retrospective study based on hospital medical records. The database was established by the Department of Neurology at Yangzhou University Affiliated Hospital. Patients with Acute minor cerebral infarction diagnosed and treated at Yangzhou University Affiliated Hospital from September 2023 to October 2024 were selected. Each enrolled patient underwent a structured telephone follow-up assessment (mRS) precisely at 90 ± 7 days after their individual stroke onset date. Thus, follow-up assessments were conducted between December 2023 and January 2025. All clinical data collection and database locking were completed in 2025. Inclusion criteria: (1) Adults aged 18–90 years, regardless of gender; (2) mRS score ≤ 2 upon admission, patients with first-time cerebral infarction or patients with a history of cerebral infarction who have new-onset cerebral infarction; (3) Completion of polysomnography. Exclusion criteria: (1) Exclusion of patients with Restless Legs Syndrome (RLS), Rapid Eye Movement Sleep Behavior Disorder (RBD), etc.; (2) Exclusion of patients who have received interventions for PLMS (such as dopaminergic drugs, iron supplements, etc.) or rehabilitation treatment plans after stroke; (3) Acute or non-acute cerebral infarction with NHISS ≥ 5, history of acute cerebrovascular accident, brain tumor, head injury, or spinal cord injury; (4) Severe limb paralysis unable to cooperate, severe and unstable conditions (congestive heart failure, r0065spiratory failure, liver failure, or end-stage renal disease) or comorbidities with other central nervous system diseases (Parkinson’s disease, dementia, or mental disorders); (5) Patients with dementia or severe cognitive impairment; (6) Patients with incomplete or missing information on certain scales. This study was approved by the Ethics Committee of Yangzhou University (Ethics 2023-YKL09), and all patients provided informed consent.

### 2.2 Demographic and clinical data

Collect basic information about patients from the medical record system, including gender, age, BMI, medical history (hypertension, diabetes, coronary heart disease, etc.), smoking and drinking history. At the same time, collect laboratory biochemical parameters, including white blood cell count, C-reactive protein, lipid levels, HCY, creatinine, VB12, glycosylated hemoglobin, and the NHISS score of patients upon admission.

### 2.3 Sleep-related scale assessment

#### 2.3.1 PSQI

The Pittsburgh Sleep Quality Index (PSQI) was used to assess sleep quality over the past month.

It has shown good internal consistency and construct validity in differentiating poor and good sleepers (Cronbach’s alpha = 0.83; Sensitivity 89.6%, specificity 86.5% for poor sleepers) (13).

#### 2.3.2 ESS

The Epworth Sleepiness Scale (ESS) was used to assess the presence of daytime sleepiness. The ESS is a reliable and valid measure of daytime sleep propensity, with good internal consistency and test–retest reliability (Test–retest *r* = 0.82; Correlated with MSLT: *r* = −0.42, *p* < 0.001) (14).

#### 2.3.3 HADS

The Hospital Anxiety and Depression Scale (HADS) is widely used in medical settings and has well-established psychometric properties, including good internal consistency and discriminant validity for screening anxiety (HADS-A) and depression (HADS-D) (15).

#### 2.3.4 MoCA

The Montreal Cognitive Assessment (MoCA) is used to evaluate cognitive function with good sensitivity, specificity and test–retest reliability. These include visuospatial ability, naming, attention, language, abstraction, delayed recall, and orientation (ICC = 0.92; Sensitivity 89% for vascular cognitive impairment) (16).

#### 2.3.5 SCD-Q9

The SCD-Q9 (Subjective Cognitive Decline Questionnaire) is a brief screening tool for subjective cognitive complaints, showing good feasibility and associations with objective cognitive measures (*α* = 0.85; Correlated with MMSE: *r* = 0.52, *p* < 0.001) (17).

All of the above scales are well-developed, standardized instruments that have demonstrated reliability and validity in both general and clinical populations, including stroke patients.

### 2.4 Polysomnography

The included patients have all undergone monitoring using the SOMNOmedics V6 polysomnography device, which includes the following neurophysiological indicators: six-channel electroencephalogram, two-channel electrooculogram, two-channel electromyogram of the jaw, electrocardiogram, nasal airflow, snoring microphone, chest and abdominal movement, blood oxygen saturation, and leg movement events. According to The World Association of Sleep Medicine (WASM) standards (18), leg movement events are manually analyzed by three professional polysomnographic technologists. If the interpretation results differ, the three technologists will discuss and decide together. Leg movement events are represented by the periodic limb movement index (PLMI), which indicates the frequency of periodic leg movements occurring per hour, with each movement lasting 0.5 to 1.0 s and an interval of 5.0 to 90.0 s. The occurrence of three or more movements is considered one instance of periodic limb movement.

### 2.5 Neuroimaging

All patients underwent neuroimaging examinations in the radiology department of our hospital, and MR images were obtained using a 3.0T MR scanner. The locations of the infarct lesions were collected, including the cortex, basal ganglia, centrum semiovale, corona radiata, thalamus, cerebellum, and brainstem.

### 2.6 Neurological function assessment

The baseline National Institutes of Health Stroke Scale (NIHSS) score at the time of admission was collected to assess the degree of baseline neurological deficit.

The modified Rankin Scale (mRS) was assessed via structured telephone interview at a target timepoint of 90 ± 7 days post-stroke onset. To ensure rating consistency and minimize inter-rater variability, all participating neurologists completed a certified online mRS training and certification module prior to study commencement. Furthermore, any cases with ambiguous functional status during the telephone interview were independently adjudicated by a third senior neurologist blinded to the initial assessment. Actual follow-up intervals (defined as days from stroke onset to mRS assessment) were recorded for all patients. As shown in Supplementary Table 1, the median follow-up time was 91 days (IQR: 90–94 days). This narrow window minimizes differential misclassification bias. The 3-month follow-up was selected based on established evidence that spontaneous neurological recovery plateaus by 90 days in minor cerebral infarction patients, with minimal functional gains thereafter. This is consistent with the AHA/ASA guideline recommendation that assessing outcomes at this critical time point captures the greatest potential for recovery before chronic disability has stabilized (19). The mRS Scale is a widely used and clinically validated measure of global disability and functional outcomes after stroke. It assesses the degree of dependence in daily activities on a scale ranging from 0 (no symptoms) to 6 (death). A score of 0–2 indicates functional independence (good outcome), while a score of 3–5 indicates varying degrees of disability requiring assistance, and 6 indicates death (20). Based on the scoring results, follow-up participants were grouped into a good prognosis group (0–2 points) and a poor prognosis group (3–6 points). All scores were completed by more than two experienced senior neurologists.

### 2.7 Statistical analysis

Statistical analysis was performed using SPSS 26.0 (IBM Corp., Armonk, United States). All data were first subjected to normality and homogeneity of variance tests. Normally distributed numerical data were expressed as mean ± standard deviation (SD), while skewed numerical data were expressed as median and interquartile range (IQR). Intergroup comparisons were made using independent samples *t*-test or rank-sum test. Categorical data were expressed as percentages, and intergroup comparisons were conducted using chi-square tests. The correlation between sleep-related scales and polysomnography with prognosis was analyzed using Spearman correlation analysis. Benjamini–Hochberg false discovery rate (FDR) correction was applied in the correlation analysis to control for type I error inflation. All *p*-values reported by Spearman correlation analysis are FDR-corrected *q*-values. Further, indicators related to prognosis (data with *p* < 0.05 in correlation analysis) along with age, hypertension, cholesterol, and brain infarction in the corona radiata were included in a binary logistic regression analysis to assess factors affecting the prognosis of patients with acute mild stroke. All statistical tests were two-sided, showing odds ratios (OR) with 95% confidence intervals (CI), and *p* < 0.05 was considered statistically significant.

Although mRS is ordinal, we selected Spearman correlation because: (1) It is the most widely used method for stroke prognosis studies(21); (2) Our primary predictors (PLMI) is continuous; (3) Simulation studies show minimal difference between Spearman and Kendall for n > 200 (22).

## 3 Results

### 3.1 Demographic and clinical data

A total of 766 patients were included, with older age, a history of hypertension, and higher HCY levels associated with poorer prognosis (*p* = 0.003; *p* < 0.001; *p* = 0.047; Table 1). There were no statistically significant differences between the two groups in terms of gender, BMI, medical history (hypertension, diabetes, coronary heart disease, etc.), smoking and drinking history, and biochemical indicators (white blood cell count, C-reactive protein, lipid levels, creatinine, VB12, and glycated hemoglobin) (all *p* > 0.05).

**Table 1:** Demographic and clinical data.

Characters	mRS ≤ 2 (*n* = 563)	mRS ≥ 3 (*n* = 203)	*t*/*z*/*χ*^2^	*p*
Age, years	59.38 ± 15.69	63.12 ± 14.891	−2.956	0.003
BMI, g/m^2^	25.46 ± 3.91	25.85 ± 3.88	−1.238	0.218
Male, %	358 (63.6)	130 (64.0)	0.013	0.909
Smoking, %	146 (25.9)	64 (31.5)	2.347	0.126
Drinking, %	149 (26.5)	54 (26.6)	0.001	0.970
Hypertension, %	308 (54.7)	144 (70.9)	16.246	<0.001
Diabetes mellitus, %	154 (27.4)	65 (32.0)	1.591	0.207
Atrial fibrillation, %	32 (5.7)	10 (4.9)	0.165	0.684
Coronary artery disease, %	65 (11.5)	18 (8.9)	1.108	0.293
WBC,10^9^/L	6.76 ± 2.26	6.69 ± 2.15	0.372	0.710
CRP, mg/L	1.54 (0.50, 4.20)	1.38 (0.51, 4.13)	−0.040	0.968
TG, mmol/L	1.00 (1.43, 2.09)	1.46 (1.01, 2.11)	−0.329	0.742
TC, mmol/L	4.09 ± 1.24	3.93 ± 1.04	2.472	0.072
LDL-C, mmol/L	2.55 ± 1.03	2.41 ± 0.87	1.921	0.055
HDL, mmol/L	1.23 ± 0.55	1.21 ± 0.42	0.351	0.726
Hcy, μmol/L	9.83 (8.35,12.70)	10.60 (8.00, 14.30)	−1.983	0.047
SCR, μmol/L	71.97 ± 28.97	78.03 ± 59.71	−1.876	0.061
VB12, pmol/L	354.00 (254.00, 485.00)	326.00 (218.00, 462.00)	−1.418	0.156
HbA1c, %	6.49 ± 1.59	6.57 ± 1.41	−0.579	0.563

BMI, body mass index; TG, triglyceride; TC, triglyceride; LDL-C, Low-density lipoprotein C; HDL, high dens ity lipoprotein; Hcy, homocysteine; SCR, serum creatinine; HbA1c, glycosylated hemoglobin; WBC, leukocyte; CRP, C-reactive protein; VB12, Vitamin B12.

### 3.2 Comparison of sleep-related scales and PSG results

Patients in the poor prognosis group had higher PSQI scores (*p* = 0.003, Table 2), while no significant abnormalities were observed in ESS, HADS, MoCA, and SCD-Q9 scores between the two groups (all *p* > 0.05). Table 3 presents a comparison of PSG results between the two groups, indicating that patients with poor prognosis had longer total sleep time (*p* = 0.014), lower sleep efficiency (*p* = 0.022), longer proportion of N1 sleep (*p* < 0.001), higher AHI (*p* = 0.020), and higher PLMS-related awakening index (*p* = 0.004) and PLMI (*p* = 0.001). No differences were found between the two groups for other indicators.

**Table 2:** Comparison of sleep-related scales.

Characters	mRS ≤ 2 (*n* = 563)	mRS ≥ 3 (*n* = 203)	*t*/*z*/*χ*^2^	*p*
ESS	6.00 (3.00, 12.00)	7.00 (3.00, 11.00)	−0.799	0.436
HADS(A)	2.00 (0.00, 5.00)	2.00 (0.00, 4.00)	−0.554	0.580
HADS(D)	2.00 (0.00, 4.00)	2.00 (0.00, 4.00)	−0.242	0.809
MoCA	20.58 ± 5.76	21.17 ± 4.79	−1.410	0.159
PSQI	8.71 ± 4.35	9.48 ± 4.69	−2.133	0.033
SCD-Q9	4.00 (2.00, 6.00)	4.00 (2.00, 6.00)	−0.248	0.804

**Table 3:** Comparison of PSG results.

Characters	mRS ≤ 2 (*n* = 563)	mRS ≥ 3 (*n* = 203)	*t*/*z*/*χ*^2^	*p*
TST, min	391.26 ± 121.57	366.66 ± 122.60	2.466	0.014
SE, %	78.23 ± 23.36	74.12 ± 16.67	2.300	0.022
WASO, min	64.40 (35.75, 130.85)	92.85 (43.00, 144.65)	−1.615	0.107
SOL, min	8.10 (3.50, 16.00)	8.80 (3.30, 19.80)	−0.196	0.845
REM, %TST	17.60 (11.30, 24.00)	16.60 (10.20, 25.20)	−0.012	0.990
N1, %TST	4.65 (2.50, 9.93)	6.70 (2.90, 15.00)	−3.530	<0.001
N2, %TST	59.80 (5.00, 21.20)	59.14 ± 21.50	0.140	0.888
N3, %TST	12.20 (5.00, 21.20)	10.00 (8.90, 45.80)	1.181	0.238
AHI	18.30 (8.90, 34.90)	22.70 (8.90, 45.80)	−2.338	0.020
Longest duration of apnea, sec	46.10 (25.00, 80.00)	49.00 (28.50, 38.40)	−1.197	0.232
Longest hypoventilation, sec	89.51 ± 29.16	93.12 ± 31.29	−1.473	0.141
Mean-SpO2, %	94.08 ± 6.34	94.61 ± 2.50	−1.148	0.251
Min-SpO2, %	84.33 ± 9.03	84.97 ± 7.28	−0.909	0.364
ArI	29.00 (17.70, 39.63)	27.80 (17.60, 28.40)	0.686	0.493
RAI	3.40 (1.30,8.20)	4.60 (2.00,10.60)	−0.505	0.613
PLMAI	1.20 (0.30,3.10)	2.10 (0.40,4.85)	−2.911	0.004
Blood pressure elevation index	11.15 (4.40, 22.70)	12.75 (5.18, 28.40)	−1.455	0.146
PLMI	8.70 (2.50, 25.80)	23.00 (6.30, 41.65)	−3.336	0.001

TST, total sleep time; SE, Sleep efficiency; WASO, wake time after sleep onset; REM, rapid eye movement sleep; SOL, sleep onset latency; AHI, apnoea–hypopnea index; Min-SpO2, minimum pulse oxygen saturation; ArI, arousal index; RAI, respiratory effort-related arousal index; PLMAI, periodic leg movement-related arousal index.

### 3.3 Comparison of neuroimaging results

As shown in Table 4, patients with poor prognosis are more likely to have infarction located in the watershed area (*p* = 0.017). There are no statistically significant differences in other imaging locations between the two groups of patients (*p* > 0.05).

**Table 4:** Comparison of neuroimaging results.

Characters	mRS ≤ 2 (*n* = 563)	mRS ≥ 3 (*n* = 203)	*t*/*z*/*χ*^2^	*p*
Cortex	103 (18.3%)	35 (17.2%)	0.112	0.738
Basal ganglia	229 (40.7%)	72 (35.5%)	1.696	0.193
Internal capsule	166 (29.5%)	46 (22.7%)	3.472	0.062
Corona radiata	178 (31.6%)	83 (40.9%)	5.708	0.017
Thalamus	157 (27.9%)	59 (29.1%)	0.102	0.749
Cerebellum	35 (6.2%)	15 (7.4%)	0.336	0.562
Brainstem	30 (5.3%)	8 (3.9%)	0.609	0.435

### 3.4 Correlation analysis of prognosis in patients with acute minor cerebral infarction

**Figure 1:**
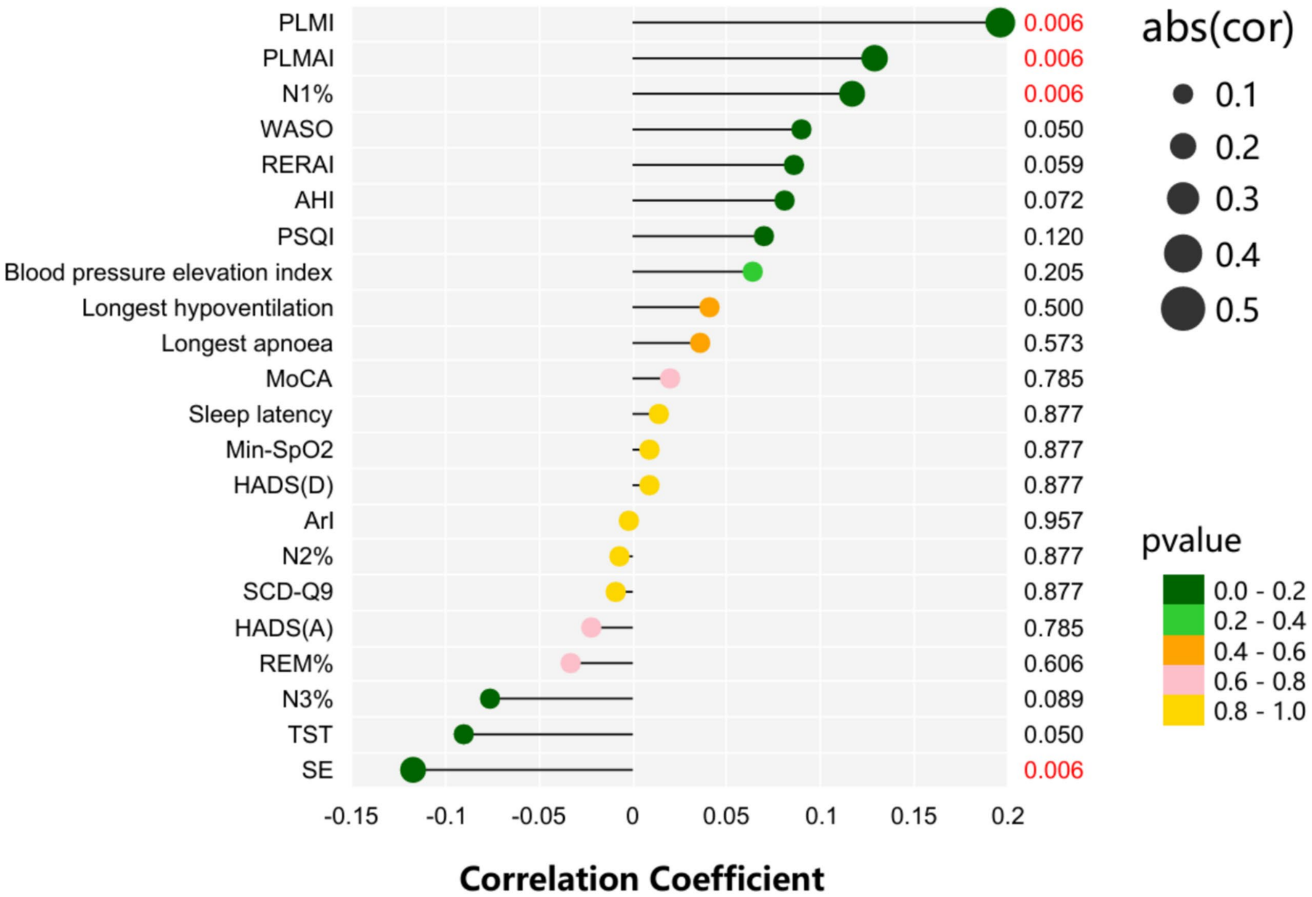
Correlation analysis of prognosis in patients with acute minor cerebral infarction.

Figure 1 shows the correlation analysis between sleep-related scales and PSG results and the prognosis of patients. We have applied the Benjamini-Hochberg false discovery rate (FDR) correction to all Spearman correlation analyses. The results indicate that N1% (*r*_S_ = 0.117, *p* = 0.006), PLMSAI (*r*_S_ = 0.129, p = 0.006), and PLMI (*r*_S_ = 0.196, *p* = 0.006) are positively correlated with prognosis, while SE (*r*_S_ = −0.117, *p* = 0.006) is negatively correlated with prognosis.

### 3.5 Binary logistic regression of mRS scores in acute minor cerebral infarction was conducted

**Figure 2:**
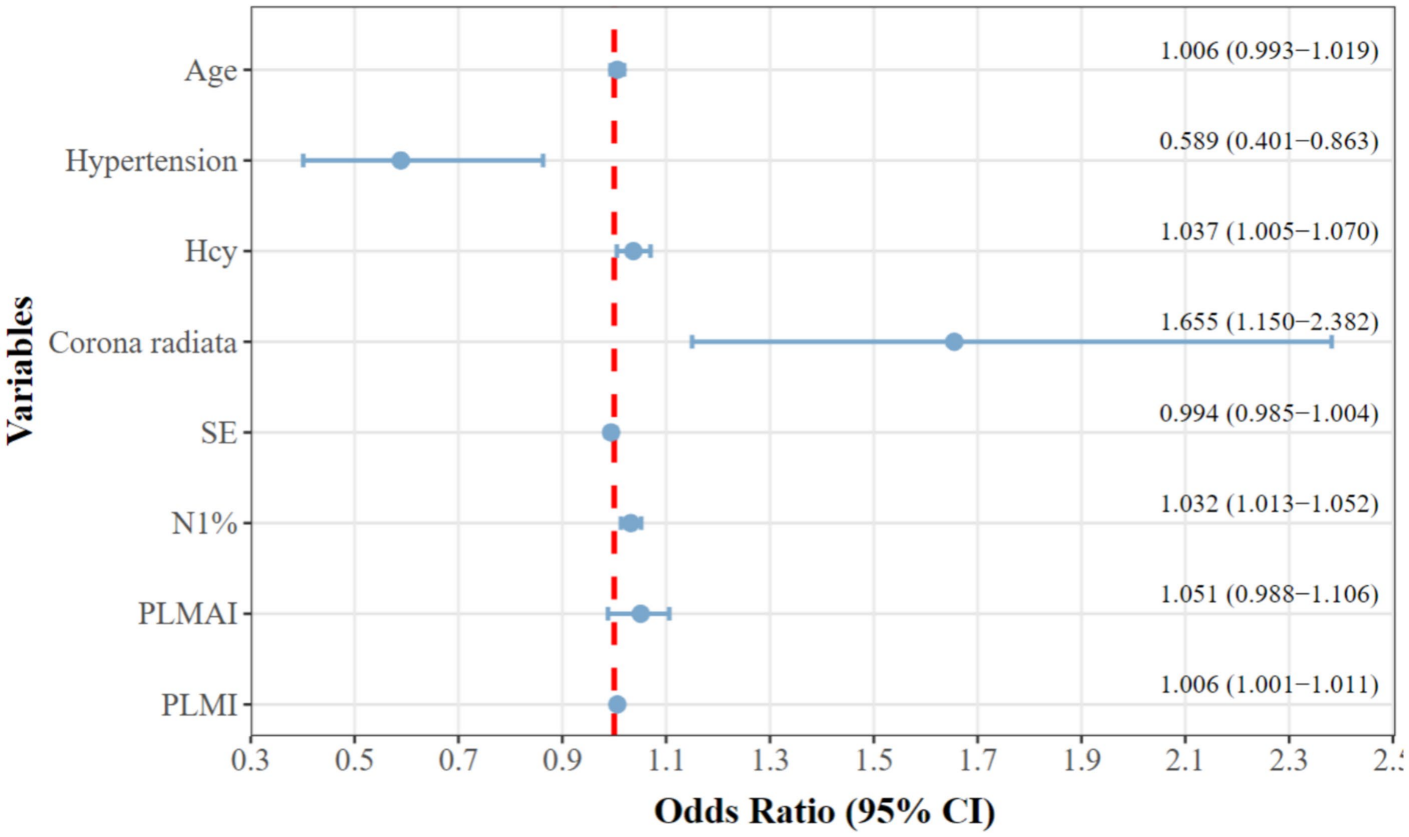
Forest plot of independent risk factors for poor prognosis.

Indicators with *p* < 0.05 from the correlation analysis, along with age, hypertension, HCY, and radiate crown area cerebral infarction, were included as covariates, with mRS grouping as the dependent variable (Table 5). The results indicated that patients with a history of hypertension (OR = 0.589, 95% CI = 0.401 to 0.863), higher HCY levels (OR = 1.037, 95% CI = 1.005 to 1.070), radiate crown area cerebral infarction (OR = 1.655, 95% CI = 1.150 to 2.382), longer N1% (OR = 1.032, 95% CI = 1.013 to 1.052), and higher PLMI (OR = 1.006, 95% CI = 1.001 to 1.010) are risk factors for the prognosis of Acute minor cerebral infarction patients at 3 months (*p* < 0.05). To enhance clinical interpretability, Figure 2 presents a forest plot of the adjusted odds ratios from the binary logistic regression. Although all five factors are risk factors for poor outcome, PLMI was a novel and modifiable target. Systematic sleep monitoring (e.g., polysomnography) in patients with minor acute stroke can identify high-risk groups for early PLMS intervention, which may improve long-term neurologic recovery.

**Table 5:** Binary logistic regression of mRS scores in acute minor cerebral infarction was conducted.

Outcome variable	*B*	S. E.	Wald	df	*p*-value	OR	95%CI for OR	
							Lower limit	Upper limit
Age	0.006	0.006	0.799	1	0.371	1.006	0.993	1.019
Hypertension	−0.530	0.195	7.362	1	0.007	0.589	0.401	0.863
Hcy	0.037	0.016	5.265	1	0.022	1.037	1.005	1.070
Corona radiata	0.504	0.188	7.365	1	0.004	1.655	1.150	2.382
SE	−0.006	0.005	1.278	1	0.258	0.994	0.985	1.004
N1%	0.032	0.010	11.075	1	0.001	1.032	1.013	1.052
PLMAI	0.049	0.026	3.560	1	0.059	1.051	0.988	1.106
PLMI	0.006	0.003	5.354	1	0.021	1.006	1.001	1.011

To assess the robustness of our primary findings against potential variations in the precise timing of the 90-day assessment window. We performed a pre-specified sensitivity analysis. We restricted the analysis to patients assessed within a narrower, more stringent window of 85–95 days post-stroke (*n* = 734, excluding *n* = 32 outside this range). This sub-analysis confirmed that a higher PLMI remained significantly associated with poor prognosis (adjusted OR = 1.006, 95% CI = 1.001 to 1.011, *p* = 0.032; see Supplementary Table 2), supporting the stability of the association even when focusing on assessments conducted closest to the theoretical peak of recovery.

## 4 Discussion

Acute mild stroke patients account for about one-third of ischemic cerebral infarction and are characterized by recurrence (23, 24). This study found that a history of hypertension, elevated HCY levels, cerebral infarction in the radiate crown area, longer N1%, and higher PLMI are risk factors for the prognosis of acute mild stroke patients.

The results of this study indicate that a history of hypertension and higher HCY levels can affect the prognosis of acute mild stroke. These factors may act through various mechanisms, exacerbating microvascular lesions in the brain, leading to poor prognosis. Hypertension, as an important risk factor for cardiovascular and cerebrovascular diseases, has the following mechanisms of damage to cerebral blood vessels. Increased blood pressure can lead to the proliferation and hypertrophy of vascular wall cells, causing cerebral vascular spasm (25). Long-term hypertension may lead to vascular stenosis or even occlusion, resulting in insufficient blood supply to brain tissue, which is associated with poor outcomes in stroke. A study on the relationship between plasma homocysteine (tHCY) levels and atherosclerotic carotid plaques and lacunar infarcts in an Asian population indicated that higher tHCY levels are associated with increased severity of carotid atherosclerotic plaques and widespread lacunar infarcts (26). This is consistent with our results, as accumulated HCY can damage the endothelial cells of small brain vessels, leading to endothelial dysfunction, apoptosis, breakdown of intercellular tight junctions, and chronic perfusion insufficiency, which is detrimental to the prognosis of cerebral infarction patients (27, 28). However, another study on elderly patients with acute cerebral infarction found that elevated tHcy at admission could not serve as a predictor for functional outcomes at 3 months and 1 year after stroke onset (29). Therefore, the relationship between HCY and neurological function in cerebral infarction patients still requires further research and validation.

Previous studies have explored the impact of infarcts in different brain regions on prognosis. The corona radiata, as a key white matter pathway connecting the cortex and basal ganglia, when damaged, can lead to widespread disruption of neural networks. One study assessed the Modified Rankin Scale 1 month after an acute cerebral infarction, using early MRI imaging on days 2 to 3 post-onset, and found that patients with infarcts in the corona radiata had poorer recovery (30). Another study reached a similar conclusion, indicating that infarcts in the corona radiata often present with more severe symptoms and worse outcomes, consistent with our findings (31). After an infarct in the corona radiata, the disruption of white matter integrity makes it more likely to experience executive function deficits and delayed motor recovery. Additionally, this area receives blood supply from deep penetrating arteries, which are more susceptible to fluctuations in blood pressure. PLMS is often accompanied by arousal and autonomic nervous activation, leading to blood pressure variability, which poses greater challenges for functional recovery after cerebral infarction (32).

The N1 sleep stage is the transitional phase from wakefulness to deep sleep, during which the proportion of sleep increases, indicating fragmented sleep or increased awakenings in patients. PLMS can lead to increased nighttime awakenings or micro-awakenings (33), making it difficult for patients to enter the deep sleep stage (N3 sleep). It is well known that N3 sleep promotes the circulation of cerebrospinal fluid through a lymphatic-like system, clearing neurotoxic substances such as β-amyloid (34). An increased proportion of N1 sleep weakens this process, leading to the accumulation of metabolic waste and exacerbating small vessel disease in the brain.

Our research findings clearly indicate that PLMS has an adverse effect on neurological recovery following acute mild stroke. Relevant literature also supports this view, with studies showing that increased PLMI is associated with a greater burden of cerebral small vessel disease, suggesting that periodic limb movements may be a novel potential marker for cerebral small vessel disease (35). PLMS may exacerbate neurological damage in acute lacunar infarction through multiple pathophysiological mechanisms. Firstly, PLMS is often accompanied by micro-awakenings, activating the sympathetic nervous system, leading to nighttime blood pressure fluctuations, reduced heart rate variability, and endothelial dysfunction (36). This autonomic dysregulation can worsen shear stress injury to cerebral small vessels, promoting the recurrence or expansion of cerebral infarction. Secondly, the sleep continuity disruption caused by periodic leg movements can activate the NF-κB pathway, increasing the release of pro-inflammatory factors (such as CRP, IL-6, TNF-*α*) and reactive oxygen species (ROS), damaging the blood–brain barrier and accelerating neuronal apoptosis (37, 38). Finally, increased PLMI leads to sleep fragmentation (39), weakening sleep-dependent neurorepair mechanisms such as synaptic remodeling and metabolic waste clearance, ultimately delaying functional recovery.

### 4.1 Limitations

This study has some limitations. First, the single-center design with retrospective data collection may introduce regional/population selection bias and limit generalizability. Additionally, reliance on a single PSG session cannot account for night-to-night variability in sleep parameters, potentially affecting the robustness of sleep-related associations. Second, Regarding the issue of the ordinal nature of mRS. And the possibility of side-ranking in sleep-related measures such as PSQI, while Kendall’s tau-b is a suitable non-parametric correlation measure for ordinal data and less sensitive to ties, Spearman’s correlation was chosen primarily because of its wide use in stroke outcome studies and applicability to non-normally distributed variables. Crucially, the core findings relied on binary logistic regression, which essentially addressed the conventional question and confirmed that PLMI was an independent risk factor (OR = 1.006, *p* = 0.021). The consistency of the key correlations (e.g., PLMI-mRS: *r*_S_ = 0.196, *p* = 0.006) and regression results indicates that our conclusions are robust. Third, the follow-up period was relatively short, which may not adequately reflect the potential impact of PLMS on long-term recurrence rates, cognitive function deterioration, or mortality. Finally, the assessment of neurological outcome via telephone-administered mRS, while practical for large cohorts, may introduce subjectivity and inter-rater variability despite being conducted by experienced neurologists. Although we implemented standardized training and adjudication protocols, subtle functional deficits could be underdetected in remote assessments. Future prospective studies with multi-center designs, serial PSG, in-person assessments, and extended follow-up are warranted.

## 5 Conclusion

PLMS is an independent risk factor for impaired neurological recovery in patients with acute minor cerebral infarction. This finding suggests that systematic sleep monitoring should be conducted in clinical practice for acute minor cerebral infarction, and early identification and intervention targeting PLMS may become a new focus for improving long-term prognosis in patients.

## Data Availability

The raw data supporting the conclusions of this article will be made available by the authors, without undue reservation.
