# Supplementary material for: The impact of periodic leg movements during sleep on neurological recovery in patients with acute mild cerebral infarction
Source: Front Neurol. 2025 Aug 1;16:1610871. doi: 10.3389/fneur.2025.1610871 (PMC12354370; doi:10.3389/fneur.2025.1610871)
Supplement: Supplementary file 1 [file Data_Sheet_1.PDF]

Supplementary Table 1 Actual Follow-up Intervals for mRS Assessment

	n	Median (IQR) Follow-up Days	Range (Days)	P*
Good prognosis	563	91(90,93)	84-97	0.385
Poor prognosis	203	91(90,94)	83-96	

* Independent samples Mann-Whitney U test

Supplementary Table 2 Sensitivity Analysis of Follow-up Time Impact

Model	Adjusted OR for PLMI (95% CI)	P
Primary analysis	1.006 （1.001,1.011）	0.021
Excluding outliers[1]	1.006 （1.001,1.011）	0.032
Adjusting for time gap[2]	1.006 （1.000,1.011）	0.033

[1] Excluding patients outside 85–95 days (n=32)

[2]Adding follow-up time as a covariate in logistic regression.
